# Exometabolome analysis reveals hypoxia at the up-scaling of a *Saccharomyces cerevisiae* high-cell density fed-batch biopharmaceutical process

**DOI:** 10.1186/1475-2859-13-32

**Published:** 2014-03-05

**Authors:** Zhibiao Fu, Thomas D Verderame, Julie M Leighton, Brante P Sampey, Edward R Appelbaum, Pramatesh S Patel, Juan C Aon

**Affiliations:** 1 Department of Microbial and Cell Culture Development, Research and Development, GlaxoSmithKline, 709 Swedeland Road, King of Prussia, PA 19406, USA; 2 Department of Process Engineering Manufacturing, Global Manufacturing and Supply, GlaxoSmithKline, 893 River Road, Conshohocken, PA 19428, USA; 3 Metabolon, Inc, 617 Davis Drive, Suite 400, Durham, NC 27713, USA

**Keywords:** *Saccharomyces cerevisiae*, Fermentation process, Scale-up, Mass transfer coefficient, Oxygen uptake rate, Exometabolomics, Metabolome profiling, Hypoxia

## Abstract

**Background:**

Scale-up to industrial production level of a fermentation process occurs after optimization at small scale, a critical transition for successful technology transfer and commercialization of a product of interest. At the large scale a number of important bioprocess engineering problems arise that should be taken into account to match the values obtained at the small scale and achieve the highest productivity and quality possible. However, the changes of the host strain’s physiological and metabolic behavior in response to the scale transition are still not clear.

**Results:**

Heterogeneity in substrate and oxygen distribution is an inherent factor at industrial scale (10,000 L) which affects the success of process up-scaling. To counteract these detrimental effects, changes in dissolved oxygen and pressure set points and addition of diluents were applied to 10,000 L scale to enable a successful process scale-up. A comprehensive semi-quantitative and time-dependent analysis of the exometabolome was performed to understand the impact of the scale-up on the metabolic/physiological behavior of the host microorganism. Intermediates from central carbon catabolism and mevalonate/ergosterol synthesis pathways were found to accumulate in both the 10 L and 10,000 L scale cultures in a time-dependent manner. Moreover, excreted metabolites analysis revealed that hypoxic conditions prevailed at the 10,000 L scale. The specific product yield increased at the 10,000 L scale, in spite of metabolic stress and catabolic-anabolic uncoupling unveiled by the decrease in biomass yield on consumed oxygen.

**Conclusions:**

An optimized *S. cerevisiae* fermentation process was successfully scaled-up to an industrial scale bioreactor. The oxygen uptake rate (OUR) and overall growth profiles were matched between scales. The major remaining differences between scales were wet cell weight and culture apparent viscosity. The metabolic and physiological behavior of the host microorganism at the 10,000 L scale was investigated with exometabolomics, indicating that reduced oxygen availability affected oxidative phosphorylation cascading into down- and up-stream pathways producing overflow metabolism. Our study revealed striking metabolic and physiological changes in response to hypoxia exerted by industrial bioprocess up-scaling.

## Background

The cultivation of microbial strains over-expressing recombinant or native proteins is an established technique in the biopharmaceutical industry [1]. In this sector, fermentation is normally developed and optimized at small scale in the laboratory and then scaled up for commercial production. In the transition from the small to the large scale it is crucial that the productivity and product qualities remain comparable [2,3]. When this is the case large quantities of recombinant proteins with therapeutic application expressed in microbial organisms can be produced cost-effectively at high volumetric production rate. At the large scale a number of bioprocess engineering problems arise that should be taken into account to match the values optimized at the small scale level and achieve the highest productivity and quality possible.

Among the changes involved in the up-scaling of a bioprocess are the bioreactor geometry and its volume capacity, and the physical condition of the culture medium. Nutrient feeding and aeration in large scale bioreactors are performed from the top and the bottom, respectively. The long distances inherent to large scale bioreactors cause heterogeneity in substrate and oxygen distribution. Longer mixing time and hydrostatic pressure gradients are produced by greater culture volumes which in turn affect oxygen transfer rates (OTR) [4]. The challenges brought about by the large scale also have a relevant impact on cellular physiology and metabolism. For example, cells will be exposed to excess nutrient and oxygen limitation on the top, and *vice versa* at the bottom. Indeed, spatio-temporal gradients of chemicals develop in large-scale fed-batch bioreactors [5]. For example, addition of a concentrated and viscous carbon source solution at a single point onto the top surface of the growth medium results in long mixing times, i.e. ≥ 50 s at the 20 m^3^ scale [6]. Studies using computational fluid dynamics have shown that considerable glucose gradients are expected when a standard 500 g/L glucose solution is fed into a 22,000 L bioreactor [7]. These findings have received experimental confirmation, and it has been further shown that cells were frequently exposed to peak glucose concentrations in the addition zone that were several times higher than the mean [5,8] causing changes in cell metabolism, productivity and product quality [9-13]. Unlike large reactors, small laboratory bioreactors exhibit low mixing times (≤ 5 s) without significant spatio-temporal variations [14].

Metabolic/physiological responses of cells to environmental heterogeneity can be monitored by highly sensitive “-omics” technologies. Using transcriptomics and proteomics it has been shown that, under poor mixing conditions, *Escherichia coli* cells respond by increasing transcription and expression of a number of stress proteins that produce inhibition of DNA replication initiation, and reduction in rRNA synthesis and protein production in addition to reduced glycolytic activity, DNA metabolism, and synthesis of structural components [5,15]. Recently, a systematic study based on exometabolomics of four biotechnologically relevant model organisms, *E. coli*, *Bacillus licheniformis*, *Saccharomyces cerevisiae*, and *Corynebacterium glutamicum* has been reported [16]. Time-dependent effects on metabolite excretion and uptake under conditions of metabolic overflow happened presumably mediated by transport mechanisms but not from cell lysis or sampling. These results are in agreement with the idea that excreted metabolites (exometabolome) reflect the metabolic state of the cell population [16].

In the present work we address the fundamental question of how the physiological and metabolic behavior of a *S. cerevisiae* strain producing a recombinant protein changes in response to the transition from laboratory to industrial scale, 10,000 L. Specifically, we utilize exometabolomics to determine activation/inactivation of metabolic pathways and how they affect important physiological variables such as specific biomass and product yields. Insightfully, these analyses allow us to infer specific links between cell physiology and bioprocess performance at the manufacturing scale. Our studies describe how a microorganism metabolism/physiology is impacted by the up-scaling of an industrial fermentation process based on a high-cell density fed-batch culture. Furthermore, we also show that the strategy described here to approximate the large scale OTRs to those measured at lab scale can be used to effectively control the process and to ensure process robustness and reproducibility in productivity and product quality.

## Results

### Performance of the optimized fermentation process at the small scale

**Figure 1:**
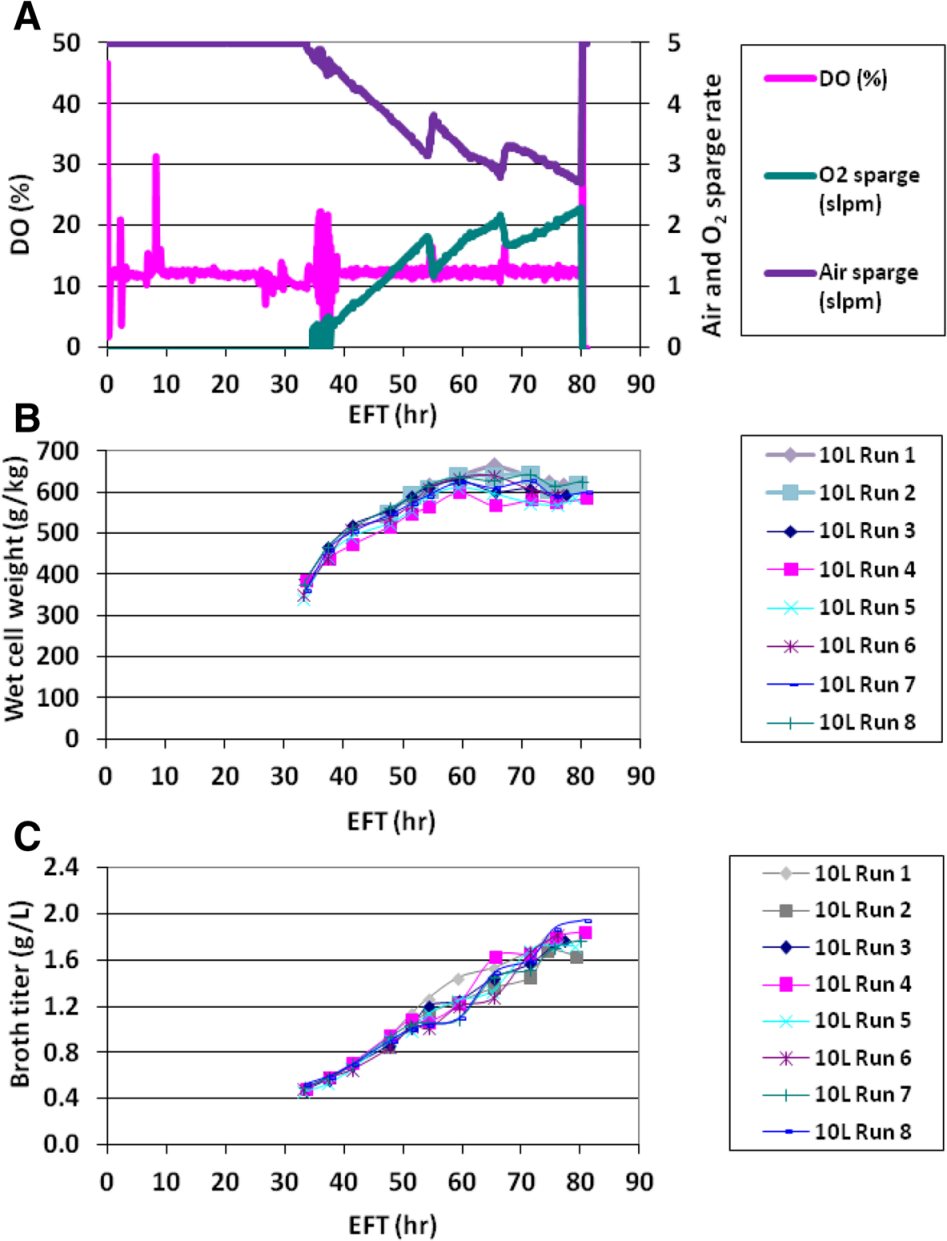
**Performance of fermentation consistency runs at the small scale (10 L) bioreactor.** Run 1–8 were consistency runs carrying out at DO 12.5%. **(A)** Online control of process parameters, DO, air sparge rate and oxygen sparge rate, for Run 1. **(B)** Wet cell weight (WCW) profiles for the 8 consistency runs. **(C)** Broth titer of the recombinant therapeutic protein profiles for the 8 consistency runs. EFT stands for the elapsed fermentation time.

In previous work, we reported the optimization of a *S. cerevisiae* high-cell density fed-batch process using a Bayesian approach [17]. The set points for critical process parameters (CPPs), temperature, pH and dissolved oxygen (DO), were selected based on the high probability region calculated to meet the acceptance criteria of relevant attributes for process performance and product quality. The selected set points for DO, temperature, and pH were 12.5%, 29°C, and 5.75, respectively. To demonstrate the process performance consistency, the temporal profiles of different attributes from eight consistency batches running at the small scale (10 L) were compared. The online process parameter profiles shown in Figure 1A are the typical profiles seen for the optimized process. Temperature and pH were well controlled at the set points (data not shown). The DO was also well controlled at the set point of 12.5% with the pure oxygen supplementation after reaching the maximum agitation. The highest pure oxygen sparge demand was determined to be less than 50% of the total capacity of 5 standard liters per minute (slpm) by the end of run (EOR) at elapsed fermentation time (EFT) 80 hr.

Figure 1B shows the wet cell weight (WCW) accumulation as one of the measurements of biomass or relative volume occupied by cells (also known as volume fraction *ϵ*_*X*_ of cell suspensions), and the consistency of those biomass accumulation profiles among the eight runs. The WCW increased to around 620 g/kg at approximately EFT 60 hr and leveled off to ~600 g/kg by the EOR. The product accumulation profiles were also consistent with a sustained increase of product concentration (measured as broth titer) up to the maximum of approximately 1.8 g/L by the EOR (Figure 1C). The quality attributes were also highly comparable between the batches (see Table four in [17]). The results indicated that the process had good controllability for each of the CPPs, and a consistent performance, and hence was ready for scale-up.

### Loss of dissolved oxygen control at the large scale

**Figure 2:**
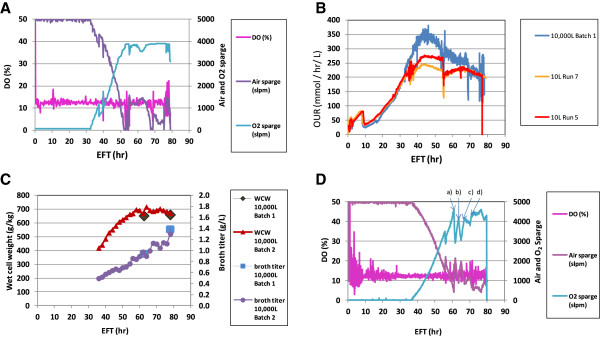
**Up-scaling the fermentation process at the large (10,000 L) scale.** Batch 1 and 2 were scaled-up and run at DO 12.5% at the large (10,000 L) scale bioreactor. Run 5 and 7 were consistency runs at DO 12.5% at the 10 L scale bioreactor. **(A and D)** Online control of process parameters, DO, air sparge rate and oxygen sparge rate, for Batch 1 and 2, respectively. Arrows, “a”, “b” and “c”, indicate the bolus addition of salt feed solution. Arrows, “d”, indicates the bolus addition of water. Additional salt solution was gradually added until the EOR, to hold the pure oxygen sparge supply below 90% of the maximum capacity. **(B)** OUR comparison between two scales with Batch 1 and Run 5 and 7. **(C)** WCW and broth titer comparison between Batch 1 and 2 at the 10,000 L scale.

The process was scaled-up from 10 L to large scale (10,000 L) as described in “Methods” based on matching the oxygen uptake rate (OUR) exhibited at the 10 L scale, with the ultimate goal to achieve reproducibility and consistency in productivity and product quality at the 10,000 L scale. A trial run, Batch 1, at the 10,000 L scale was conducted with the same temperature, pH and DO set points as the 10 L bioreactor. The pure oxygen sparge rate (*F*_*O2*_) was set at maximum 4,000 slpm while the total gas flow (*F*_*TOT*_) was set at maximum 5,000 slpm. During the entire incubation time (EFT 80 hr) temperature and pH were well controlled at the selected set points 29°C and 5.75, respectively (data not shown). However, DO was only controlled at set point until EFT 52 hr, and started to drop because the *F*_*O2*_ reached the maximum 4,000 slpm (Figure 2A). In the attempt to re-gain DO control, the glucose feed rates were reduced to decrease the cell oxygen demand. However, a negative impact on broth titer was observed, i.e. EOR broth titer decreased from 1.8 g/L to 1.38 g/L (Figure 2C and Figure 1C). OURs were comparable between the trial Batch 1 and the 10 L scale runs 5 and 7 up to EFT 35 hr and after EFT 70 hr, but OURs were not comparable between EFT 35 hr and 70 hr as seen in Figure 2B.

Another trial run, Batch 2, at the 10,000 L scale was conducted with the same temperature, pH and DO set points as Batch 1 while the pure oxygen sparge supply capacity was increased to equalize *F*_*TOT*_ (5,000 slpm). Beyond the increase of the pure oxygen supply capacity, there were bolus additions of diluents, i.e., salt feed solution and water, to maintain DO control. The rationale was to improve the oxygen mass transfer in the culture, and to make no modifications to the glucose feed rates to avoid a drop in productivity by the EOR. The first bolus supplement of salt solution (~200 L) was added at EFT 59.4 hr over 40 minutes when the pure oxygen sparge supply reached 90% of the maximum capacity (“a” arrow, Figure 2D). The second addition of salt solution (~200 L) was completed over about 90 minutes when the pure oxygen sparge supply reached 82% of the maximum capacity at EFT 62.4 hr (“b” arrow, Figure 2D). The third salt solution addition (~200 L) was started at EFT 65.4 hr to last about 3.5 hrs to hold the pure oxygen sparge supply around 80% of the maximum capacity (“c” arrow, Figure 2D). The next addition was water (~127 L) over about 30 minutes at EFT 69.9 hr (“d” arrow, Figure 2D), but oxygen supply rate did not drop as seen with the three previous salt solution additions. After EFT 70.9 hr additional salt solution was gradually added until the EOR, to hold the pure oxygen sparge supply below 90% of the maximum capacity. Consequently, the amount of salt solution added was 4.4 times the intended amount. The WCW increased to ~700 g/kg at EFT 60 hr, leveled off during the additions of salt solution, and finally decreased to ~670 g/kg by the EOR (Figure 2C). Nevertheless, product titer kept on increasing during the entire fermentation, albeit, at a slower pace (Figure 2C). The EOR broth titer was 1.3 g/L which was marginally decreased with respect to Batch 1.

In summary, control on the DO process parameter was improved by the additions of water and salt solution and pure-oxygen supply capacity increase without modifications to the profile of glucose feed rates. The process productivity was still lower than that at the 10 L scale bioreactor.

### Differences in mass transfer coefficient, cell relative volume, and apparent viscosity introduced by scale-up

**Figure 3:**
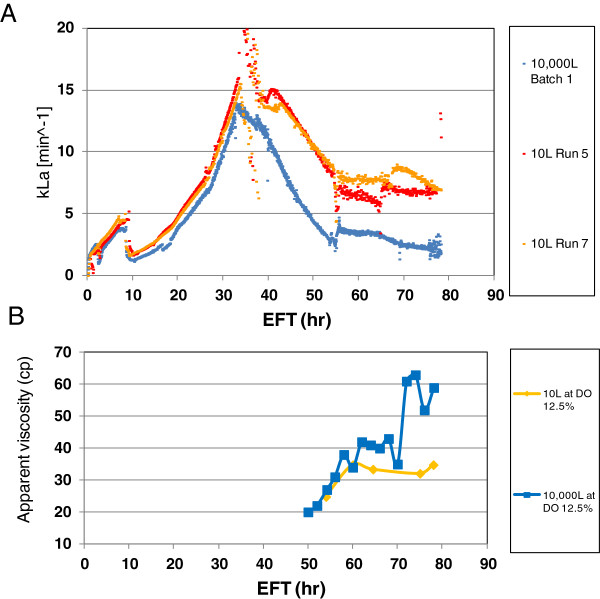
**Mass transfer coefficient and culture apparent viscosity assessment.** Batch 1 was scaled-up and run at DO 12.5% at the large (10,000 L) scale bioreactor. Run 5 and 7 were consistency runs at DO 12.5% at the 10 L scale bioreactor. **(A)** Mass transfer coefficient (*k*_*L*_*a*) comparison between two scales. **(B)** Apparent viscosity comparison. The apparent viscosity for the 10,000 L scale Batch 2 and one parallel at 10 L scale run at DO 12.5% was measured at 750 s^−1^ shear rate as described in “Methods”.

To evaluate the causes of higher pure oxygen sparge demand in the 10,000 L scale bioreactor, we compared the mass transfer coefficient (*k*_*L*_*a*) from both scales. As seen in Figure 3A, *k*_*L*_*a* initially increased as the agitation speed was ramped up from approximately EFT 10 to 34 hr, but then decreased during the constant maximum agitation speed irrespective of increasing the supply of oxygen-enriched air. The *k*_*L*_*a* temporal profiles had significant changes between approximately EFT 34 and 54 hr for both scales, but more pronounced at the 10,000 L scale (Figure 3A). After EFT 54 hr, the *k*_*L*_*a* appeared to level off at the 10 L scale, whereas *k*_*L*_*a* continued to decrease at lower pace at the 10,000 L scale. By the EOR, *k*_*L*_*a* dropped to ~2.5 min^−1^ and 6.5 min ^-1^ at the 10,000 L and 10 L scale, respectively. The net decrease in mass transfer coefficient was significantly higher at 10,000 L than at 10 L.

When the exhaust gas oxygen concentration was measured through the on-line mass spectrometry, the oxygen concentration was found to initially decrease until pure oxygen enrichment of the inlet gas started (approximately EFT 34 hr) and then continuously increased up to 80% and 30% oxygen concentration by the EOR at 10,000 L and 10 L scale, respectively (data not shown). The high oxygen concentration in the exhaust gas towards the EOR at the 10,000 L scale indicates that most of the oxygen injected to enrich the total inflowing gas was not efficiently transferred into the culture as reflected in the low mass transfer coefficient (Figure 3A).

In addition, apparent viscosity measurements of the culture medium were carried out at different time points during the fermentation process starting at the Batch 2 and thereafter 10 L scale batches. The apparent viscosity was low and close to that of water at the start of the process (data not shown), and increased to the EOR (Figure 3B). Although the apparent viscosity increased with the fermentation elapsed time for both scales, the level of increase was significantly lower at 10 L scale, particularly after EFT 60 hr. The apparent viscosities were about 34 centipoises (cP) and 59 cP by the EOR at 10 L and 10,000 L scale, respectively (Figure 3B). An additional difference detected between the two scales was the volume fraction occupied by cells measured as WCW; higher WCWs were observed at 10,000 L scale. Highest WCWs of 700 g/kg and 620 g/kg were measured at the 10,000 L and 10 L scale, respectively (Figure 1B and Figure 2C). Higher WCW and apparent viscosity may have caused the decrease in *k*_*L*_*a* observed at 10,000 L scale.

Assuming OTR = OUR at DO set point, if *k*_*L*_*a* is lower at the 10,000 L scale, then the driving force (ΔC) must be increased to maintain the DO (see “Methods”). Consequently, higher pure oxygen supply was demanded by the culture at 10,000 L scale, and the actual DO concentration in the liquid phase could be lower than the target set point due to existing oxygen concentration heterogeneities inside the large scale bioreactor. The cells were very likely exposed to lower DO concentrations. Therefore, the DO and pressure set points were changed to higher values in the 10,000 L scale as discussed in the following section.

### Performance of the fermentation process with higher dissolved oxygen at the large scale

**Figure 4:**
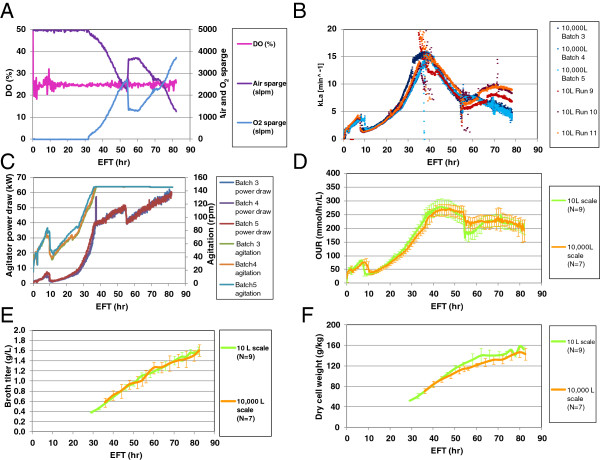
**Overall consistent fermentation performance between two scales after fine tuning adjustments.** Batch 3 was scaled-up and run at DO 25% and doubled back pressure at the 10,000 L scale bioreactor. Six more batches, Batch 4–10, were completed at the same conditions at the 10,000 L scale. Nine more runs, run 9–17, were completed in parallel with seven 10,000 L scale batches running at DO 12.5% and doubled back pressure at the 10 L scale bioreactor. **(A)** Online process parameters control for 10,000 L scale Batch 3. **(B)** Mass transfer coefficient comparison between two scales with Batch 3–5 at the 10,000 L scale and Run 9–11 at the 10 L scale. **(C)** Agitator power draw and agitation profiles for Batch 3–5 at 10,000 L scale. There was an interrupted gas flow at ~ EFT 37 hr at Batch 4, causing a spike in agitator power draw. **(D)** OUR comparison between two scales with 7 batches at 10,000 L scale and 9 runs at the 10 L scale. **(E)** Broth titer comparison between two scales with 7 batches at 10,000 L scale and 9 runs at 10 L scale. **(F)** Dry cell weight (DCW) comparison between two scales with 7 batches at 10,000 L scale and 9 runs at 10 L scale. Error bars represents “mean +/− one standard deviation”.

To investigate whether the higher DO set point by elevating the average DO concentration inside the 10,000 L scale bioreactor and higher pressure to enhance the oxygen solubility would reduce the pure oxygen sparge demand, more trial runs were carried out. One trial run, Batch 3, was kept at the same temperature and pH; but DO set point was increased to 25% and the backpressure was doubled to 10 psi. Additions of water and salt solution as in Batch 2 were also implemented in Batch 3 but added gradually into the culture together with the glucose and salt feed solution, respectively. The salt feed program was adjusted to add an extra 94% of salt solution to the intended volume before process scaling-up. Effectively the glucose feed was diluted with water by approximately 13% to cover losses by evaporation. Then the glucose feed program was adjusted after 52 hr fed-batch time to deliver the same intended amount of glucose but extended for 2 hrs. As shown in Figure 4A, DO for Batch 3 was all under control throughout the entire run. The maximum pure oxygen sparge demand was at less than 4,000 slpm (80% of the maximal capacity) by the EOR. Other process parameters such as temperature and pH were also well controlled (data not shown). Then two more batches at 10,000 L scale, Batch 4 and 5, were run in parallel with three 10 L batches, Run 9, 10, and 11, to continue the verification of process performance. When the *k*_*L*_*a* values were compared between 10,000 L scale batches, Batches 3 to 5 exhibited higher values than 5 min^−1^ up to approximately EFT 70 hr (Figure 4B), but Batch 1 values were only above that value up to approximately EFT 47 hr before the adjustments (Figure 3A). When the *k*_*L*_*a* values were compared between 10 L and 10,000 L scales, those profiles are closer than those shown in Figure 3A, indicating that implemented changes improved *k*_*L*_*a* values and so the efficiency to transfer oxygen into the medium.

To further characterize the effect of the implemented changes on the mixing of the 10,000 L bioreactor, the agitation speed and the agitator power draw throughout the fermentation time from batches 3, 4 and 5 were compared as shown in Figure 4C. Consistently in every batch, the agitator power draw increased up to 40 kW (kilowatts) in the approximately first 35 hrs following the agitation speed ramping-up as required to control the dissolved oxygen at set point. After EFT 35 hr the agitation speed remained constant at maximal of 146 rpm until EOR, but the power draw kept increasing except for the sudden drop at ~ EFT 54 hr (Figure 4C). The gassed power input per unit of culture volume was 3.85 kW/m^3^ at EFT 34 hr and 5.54 kW/m^3^ at EOR. These high power inputs, compared to those typically achieved with yeast fermentations in vessels up to 19,000 L or higher scale [12], suggest that the bioreactor is likely to be well mixed.

**Figure 5:**
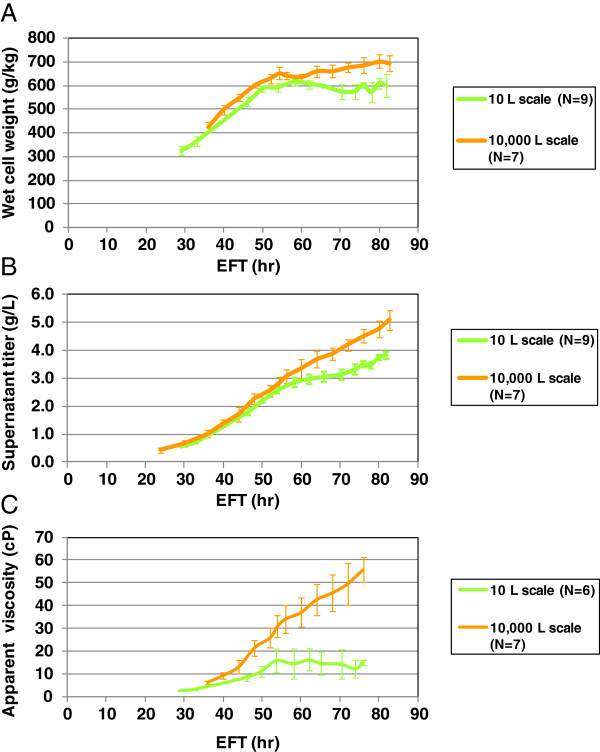
**Performance differences between two scales.** Seven batches, Batch 4–10, were completed at DO 25% and doubled back pressure at the 10,000 L scale bioreactor. Nine runs, run 9–17, were completed in parallel with seven 10,000 L scale batches running at DO 12.5% and doubled back pressure at the 10 L scale bioreactor. **(A)** WCW profile comparison between two scales. **(B)** Product titer measured at the culture supernatant profile comparison between two scales. **(C)** Culture apparent viscosity measured at shear rate of 750 s^−1^ profile comparison between scales. Only run 12–17 were measured the apparent viscosity at the 10 L scale. Error bars represents “mean +/− one standard deviation”.

To further demonstrate the consistency in cell performance, we compared the OUR values from 9 batches at the 10 L scale (runs 9 to 17), which were completed with the changes of pressure and the salt diluents and run in parallel with 10,000 L scale batches, to the 7 batches at 10,000 L scale (batches 4 to 10). As seen in Figure 4D, the OUR temporal profiles for the two scales became comparable. Interestingly, the product accumulation profiles (broth titer) (Figure 4E) as well as other qualities, purity by reverse phase-HPLC (RP-HPLC), purity by capillary iso-electric focusing (cIEF), protease activity, and product-related 6AA-impurity level by ELISA assay, (data not shown) were also comparable. The acceptance criteria for broth titer, and the product quality attributes, such as purity by RP-HPLC and cIEF, protease activity, and the 6AA-impurity, were established and the related assay descriptions are detailed in our previous work [17]. The biomass concentration profiles measured by dry cell weight (DCW) were also comparable between two scales (Figure 4F), indicating the total number of cells per unit volume were comparable even though the WCWs were slightly different (Figure 5A). Taken together, the results indicate the successful up-scaling of the process after some fine-tuning adjustments.

Nevertheless, some differences between the two scales remained, such as WCW, supernatant titer and apparent viscosity. The WCW temporal profiles were comparable before EFT 54 hr and diverged thereafter, reaching maximum values of 700 g/kg and 600 g/kg at 10,000 L and 10 L scale, respectively (Figure 5A). Interestingly, the product concentration in the spent medium (supernatant titer) showed a similar trend for the accumulation profile as the WCW (Figure 5B). When the supernatant titers were normalized with respect to its WCWs, the differences were minimized, showing a high similarity in broth titer performance (Figure 4E). The apparent viscosity temporal profiles diverged earlier at around EFT 40 hr between scales, reaching maximum values of 56 cP and 15 cP at 10,000 L and 10 L scale, respectively (Figure 5C).

### Metabolite profiling at the small and large scale cultures

To characterize the metabolic behavior of the host strain of *S. cerevisiae* at both production scales, we performed metabolomics of the extracellular medium or exometabolome as described in the “Methods”. The exometabolome represents intracellular metabolism as reflected by excreted metabolites. Under the production conditions cell viability was measured by EOR at both scales, 10 L and 10,000 L, based on the red fluorescence dye, propidium iodide (PI) assay described in the “Methods”. At both scales the yeast cell viability was maintained in average slightly above 93%. In addition, the retention of the Pr-1 protein-encoding plasmid was maintained above 95% at both small and large scales (data not shown). We focus our analysis at the two scales on membrane and energetic metabolism including the mitochondrial functionality.

### Mevalonate/ergosterol pathway and membrane metabolism

**Figure 6:**
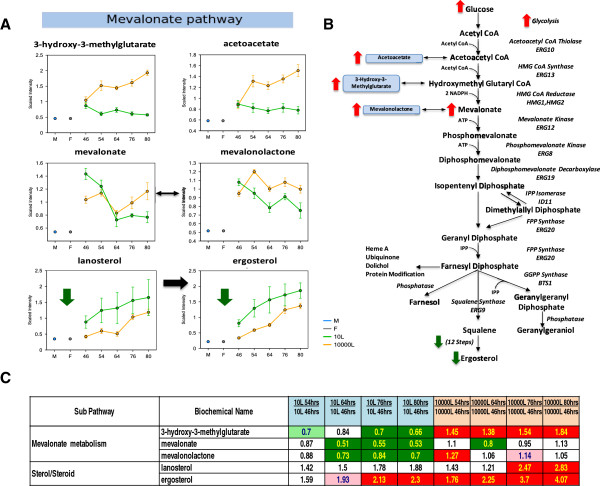
**Pathway diagram and corresponding heat map table and line plot graphs showing biochemical differences in the intermediates of the mevalonate/ergosterol pathway between scales. (A)** Line plot graphs comparison between two scales. **(B)** Pathway diagram comparison between two scales. **(C)** Heat map table comparison between two scales. Red and green arrows show changes in the 10,000 L scale with respect to that at the 10 L scale. The intermediates measurement and data analysis were determined as described in “Methods”. Error bars represent “mean +/− one standard deviation”. “M” and “F” in the time axis for every line plot stand for Medium and Feed samples, respectively.

Alterations in the mevalonate/ergosterol pathway have been shown to affect membrane composition and permeability. These effects could potentially influence the volume occupied by cells as shown in Figure 5A, or trafficking of metabolites through membranes and cell wall. Figure 6 depicts the metabolite profiles from the mevalonate/ergosterol pathway obtained at 10 L and 10,000 L production scales. Concentrations from both ergosterol and its precursor lanosterol were lower at 10,000 L than at 10 L scale, but the kinetics of increase was similar (Figure 6A). However, 3-hydroxy-3-methylglutarate and acetoacetate increased over time at 10,000 L scale while they decreased at 10 L scale (Figure 6A). The consistent pattern of elevated concentrations from upstream metabolites, 3-hydroxy-3-methylglutarate and acetoacetate, and decreased levels from downstream metabolites, lanosterol and ergosterol, lend notable credence to a diminished or impaired ergosterol synthesis at the 10,000 L scale culture (Figure 6B).

**Figure 7:**
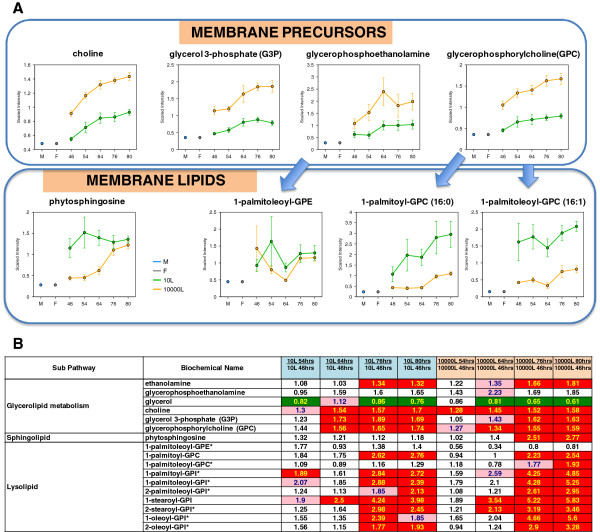
**Line plot graphs and corresponding heat map table showing the differences of the glycerolipid membrane precursors and the membrane lipids between scales. (A)** Membrane precursors and membrane lipid comparison between scales. **(B)** Heat map table comparison between scales. Blue arrows indicate relationships between precursors and expected membrane synthesis products. The intermediates measurement and data analysis were determined as described in “Methods”. Error bars represent “mean +/− one standard deviation”. “M” and “F” in the time axis for every line plot stand for Medium and Feed samples, respectively.

Concomitant to changes in ergosterol, the culture medium at 10,000 L scale exhibited a substantial and sustained increase in precursors of membrane synthesis choline, glycerol-3-phosphate (G3P), glycerophosphoethanolamine, and glycerophosphorylcholine (GPC), as compared with 10 L scale (Figure 7A). Conversely, membrane lipidic components such as phytosphingosine, 1-palmitoylglycerophosphocholine (1-palmitoyl-GPC), 1-palmitoleoylglycerophosphocholine (1-palmitoleoyl-GPC) and 1-palmitoleoylphosphoethanolamine (1-palmitoleoyl-GPE) were lower in 10,000 L than in 10 L scale (Figure 7A). These findings suggest a decreased utilization of membrane building blocks over time in agreement with the idea of a lower level of membrane biosynthesis in the 10,000 L scale cultures (Figure 7B). Throughout the fermentation, the profiles of biomass concentration based on DCW measurements were comparable between scales (Figure 4F) and the levels of precursors for membrane synthesis were significantly higher at 10,000 L than in 10 L scale. These results suggest a compromised *S. cerevisiae* cell membrane integrity. Furthermore, the compromised membrane integrity of cells at 10,000 L scale possibly explains the sustained and slow pace increase of WCW compared to 10 L scale after EFT 54 hr until EOR (Figure 5A).

### Glycolysis, tricarboxylic acid (TCA) cycle and mitochondrial dysfunction

**Figure 8:**
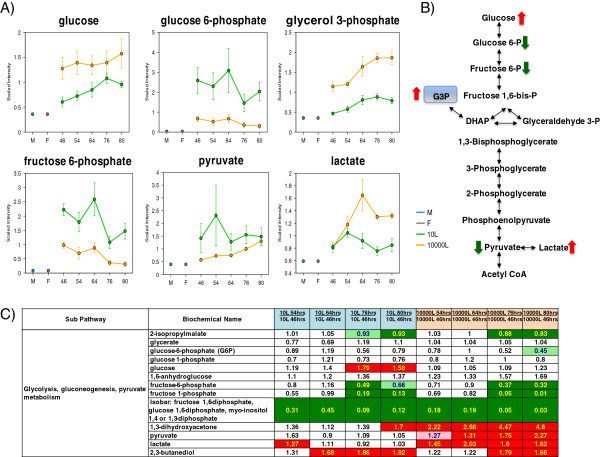
**Pathway diagram, line plot graphs and corresponding heat map table showing differences in glucose metabolism intermediates between scales. (A)** Line plot graphs comparison between two scales. **(B)** Pathway diagram comparison between two scales. **(C)** Heat map table comparison between two scales. Red and green arrows show changes in the 10,000 L scale with respect to that at the 10 L scale. The intermediates measurement and data analysis were determined as described in “Methods”. Error bars represent “mean +/− one standard deviation”. “M” and “F” in the time axis for every line plot stand for Medium and Feed samples, respectively.

The glycolysis and the TCA cycle belong to the backbone central catabolism providing energy in the form of ATP and reducing equivalents, and key intermediate precursors for the cellular biomass. These pathways are heavily influenced by the level of glucose in the medium, which was higher at the 10,000 L than the 10 L scale (Figure 8). Increased medium glucose concentration correlated with a notable decrease in glucose-6-phosphate, fructose-6-phosphate and pyruvate levels from glycolysis. In contrast, lactate increased in the 10,000 L scale until EFT 64 hr staying at higher levels than in the 10 L scale. Interestingly, glycerol-3-phosphate, a membrane lipid component that can feed into glycolysis at the level of dihydroxyacetone phosphate, was also significantly elevated in the 10,000 L scale culture medium (Figure 8). The results suggest glucose shunt to alternative degradation pathways. The sustained increase over time of erythrulose, galactitol, threitol, panose and gluconate is in agreement with activation of alternative glucose catabolism coupled to metabolite excretion to the medium likely due to metabolic overflow (Additional file 1: Figure S1). Taken together, these results indicate that a prominent difference in glucose catabolism occurs at the two scales.

**Figure 9:**
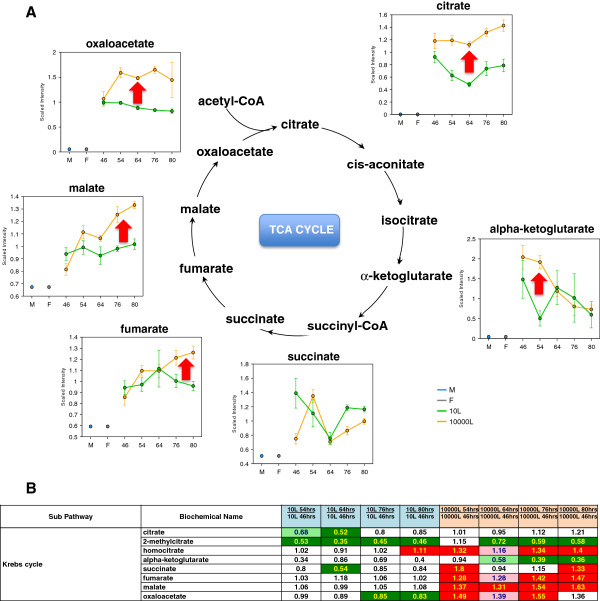
**Pathway diagram, line plot graphs and corresponding heat map table showing the differences of TCA intermediates between scales. (A)** Line plot graphs and pathway diagram comparison between two scales. **(B)** Heat map table comparison between two scales. Red and green arrows show changes in the 10,000 L scale with respect to that at the 10 L scale. The intermediates measurement and data analysis were determined as described in “Methods”. Error bars represent “mean +/− one standard deviation”. “M” and “F” in the time axis for every line plot stand for Medium and Feed samples, respectively.

The appearance of TCA cycle intermediates in the medium is considered to reflect impairment in this pathway as observed in overflow metabolism under aerobic and glucose excess condition [16]. Citrate levels were maintained at 10,000 L scale but notably diminished (significant at EFT 64 hr) at the 10 L scale (Figure 9A). Fumarate, malate and oxaloacetate increased at EFT 54 hr as compared to EFT 46 hr and kept constant or augmented over time at the 10,000 L scale. In stark contrast, most TCA intermediates were either maintained or diminished over time at the 10 L scale (Figure 9A and B). These results indicate mitochondrial dysfunction and TCA cycle overflow leading to loss of intermediates at the 10,000 L scale culture.

**Figure 10:**
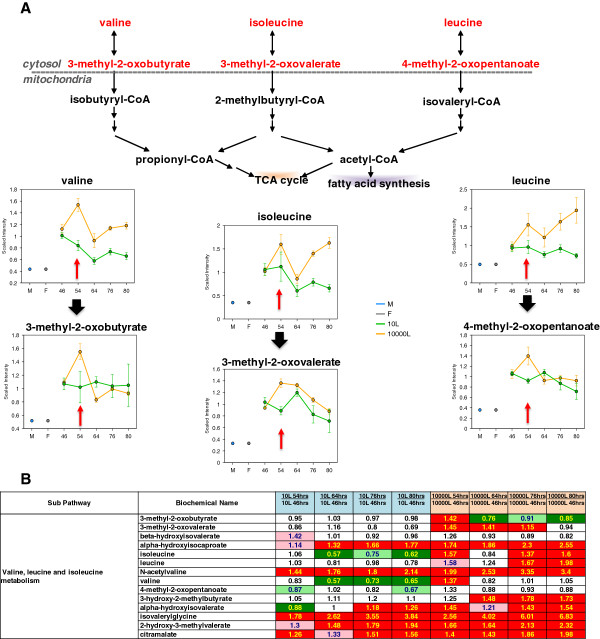
**Pathway diagram, line plot graphs and corresponding heat map table showing the changes in branch chain amino acid levels over time between scales. (A)** Line plot graphs and pathway diagram comparison between two scales. **(B)** Heat map table comparison between two scales. Red and green arrows show changes in the 10,000 L scale with respect to that at the 10 L scale. The intermediates measurement and data analysis were determined as described in “Methods”. Error bars represent “mean +/− one standard deviation”. “M” and “F” in the time axis for every line plot stand for Medium and Feed samples, respectively.

Further examination of mitochondrial function at both scales was performed through analysis of biochemical markers. An increase in branched chain amino acids, valine, isoleucine and leucine, and their alpha-keto acid intermediates are known metabolic indicators of mitochondrial dysfunction or respiratory-deficient cells [18,19]. As shown in Figure 10, branched chain amino acids (valine, isoleucine and leucine) and their alpha-keto acid intermediates (alpha-keto acids 3-methyl-2-oxobutyrate, 3-methyl-2-oxovalerate and 4-methyl-2oxopentanoate) either stayed constant or decreased over time at the 10 L scale. In contrast, branched chain amino acids were notably elevated in media at the EFT 54 hr at the 10,000 L scale. These results suggested mitochondrial dysfunction or impairment that occurred in parallel with accumulation of glycerol-3-phosphate and intermediates of TCA cycle and mevalonate pathways. Mitochondrial impairment could also reflect cell hypoxia at the 10,000 L scale culture resulting in changes in ergosterol synthesis and glucose degradation. Importantly, associated with the hypoxia phenomenon the marked differences in lanosterol and ergosterol levels found in the 10 L and 10,000 L scale media at the earliest time point (EFT 46 hr) (Figure 6A), may indicate the divergence in their metabolic behavior thus initiating the scale-specific differences unveiled by exometabolomics.

### Physiological behavior of *S. cerevisiae* and productivity at the large scale

As a result of the hypoxia experienced by the *S. cerevisiae* cells at the 10,000 L after all implemented modifications, the decrease in biomass yield on oxygen consumption, *Y*_O2_, would be an expected physiological effect. This is produced by a significant reduction of oxidative phosphorylation in mitochondria, which likely leads to functional impairment of this organelle. *Y*_O2_ is an indicator of the degree of coupling between respiration (dissimilation/catabolic pathway) and energy conservation (assimilation/anabolic pathways) in a microorganism. *Y*_O2_ often limits the maximal productivity at which a process can operate thus influencing the cost of goods. *Y*_O2_ calculated during carbon-limited growth for various organisms was found to be inversely correlated to the higher metabolite product formation rates [20]. Therefore, the lower the *Y*_O2,_ the more glucose is converted into the product instead of biomass reflecting a metabolic burden introduced by the product formation.

The average *Y*_O2_ at 10,000 L scale was 14% lower (17.5 g DCW / mol O_2_; n = 7) than that at 10 L scale (19.7 g DCW / mol O_2_; n = 9) (Table 1). The specific rate of product Pr-1 production (*q*_p_; mg Pr-1 / g Gluc/hr) and the product yield on glucose (*Y*_p_; mg Pr-1 / mol Gluc), as sole and limiting-carbon source, were also calculated. At the 10,000 L scale the average *Y*_p_ (0.75 mg Pr-1 / mol Gluc) was approximately 20% higher than at 10 L scale (0.63 mg Pr-1 / mol Gluc) (Table 1). Similarly, the *q*_p_ was 16% greater at the 10,000 L than at the 10 L scale. Taken together, these results constitute proof of principle that lower biomass yields on molecular (O_2_) oxygen produce a more efficient conversion of substrate (glucose) into product (recombinant protein). These results also reaffirm the concept that efficiency from the microorganism’s physiology/metabolism and the industrial application viewpoints are not equivalent. Consequently, we conclude that the hypoxic conditions attained in the 10,000 L scale bioreactor resulted in an increase in process productivity irrespective of detrimental metabolic and physiological effects on the host microorganism.

**Table 1:** Comparison of biomass yields, product yields and specific rate of product production between the 10 L and 10,000 L scales

**Estimated response**	**UoM**	**Average ± one std. dev**	
		**10 L scale**	**10,000 L**
		**(n=9)**	**Scale (n=7)**
*Y*_O2_	g DCW/mol O2	19.7 ± 1.4	17.5 ± 1.1
*Y*_p_	mg Pr-1/mol Gluc	0.63 ± 0.04	0.75 ± 0.05
*q*_p_	mg Pr-1/g Gluc/hr	0.043 ± 0.003	0.050 ± 0.003
*Y*_s_	g DCW/g Gluc	0.39 ± 0.02	0.39 ± 0.01

The biomass yields based on oxygen, *Y*_O2,_ are defined as amount of biomass (grams of dry cell weight (DCW)) produced per mol of molecular oxygen (O_2_) consumed by cells (g DCW / mol O_2_). The biomass yields based on carbon substrate, *Y*_s_, are defined as amount of biomass (grams of DCW) produced per gram of glucose fed (g DCW / g Gluc). The product yields based on carbon substrate, *Y*_p_, are defined as amount of secreted product (milligrams of protein Pr-1) per mol of glucose fed (mg Pr-1 / mol Gluc). The specific rate of therapeutic protein production, *q*_p_, is expressed as milligrams of product Pr-1 per gram of glucose fed and per hour of elapsed fermentation time (mg Pr-1 / g Gluc/hr). The average and one standard deviation of every estimated response indicated above come from 7 batches at 10,000 L scale, or from 9 batches at 10 L scale. UoM: unit of measurement.

## Discussion

This work describes the scale-up of a *S. cerevisiae* fermentation process from the laboratory to the industrial scale to produce a recombinant therapeutic protein. This study shows that oxygen availability is a key variable determining the successful scale-up of a fermentation process optimized at the laboratory scale. Elevations in the WCWs and culture apparent viscosity at 10,000 L scale caused the decrease of oxygen transfer efficiency. Changes of process parameters, such as dissolved oxygen and pressure set points, and salt solution addition, improved mass transfer into the culture. But some differences in above manufacturing attributes remained. Other factors, such as heterogeneities in oxygen and nutrient distribution throughout the bioreactor, should also be considered for process up-scaling [21]. These changes had a decisive impact on the metabolic pathways and the physiological behaviour of the microorganism.

### Interaction between microorganism physiology and bioprocess controllability at the large scale

The physico-chemical heterogeneities arising in a “well- mixed” large-scale bioreactor can cause microorganisms to alter their physiology in response to challenging environmental conditions [21,22]. Cell stress responses controlled by regulatory networks are activated during adaptation to suboptimal growth conditions and/or heterologous protein production [23]. However, these adaptive mechanisms can have a detrimental effect on the bioprocess related to the control and product quantity/quality in the industrial scale. In previous work we optimized the *S. cerevisiae* fermentation process at the small scale (10 L), using a multivariate Bayesian approach [17]. As a result, a robust and reproducible process at the 10 L scale bioreactor was established. Herein, we describe the scale up of that process, and characterize the differences between the small and large scale bioreactors. Decreased mass transfer coefficient and increased apparent viscosity (Figure 3) and WCW (Figure 2C) were significant at the large scale culture. Initially, we supplied higher amounts of pure oxygen sparge to maintain DO at the 10,000 L scale high-cell density culture, but the total pure oxygen supply reached its maximum capacity for Batch 1 at EFT 52 hr (Figure 2A). Increased culture apparent viscosity and WCW were two critical variables affected by the scale up (Figures 2, 3, 4 and 5). High DCW and WCW biomasses have been shown to increase the culture apparent viscosity [24,25]. Also as the viscosity of viscous streptomycete fermentation increased, the oxygen transfer coefficient (*k*_*L*_*a*) was observed to decrease [26]. From EFT 35 hr to the EOR, a three-fold decrease in *k*_*L*_*a* occurred at the large scale (Figure 4) likely due to interfacial blanketing, i.e. proteins and metabolites adsorption at gas–liquid interfaces in bubbles thus reducing gas–liquid area contact. Consequently, the lower *k*_*L*_*a* caused the low oxygen availability, creating hypoxic conditions in the culture. In addition, the culture at DO 5% exhibited higher WCW and apparent viscosity than those at DO 36.8% at the 10 L scale, provoking higher oxygen supply demand (data not shown). Regaining the DO control at the 10,000 L scale demanded setting higher DO and pressure set points together with culture dilution (Figures 2 and 4). The rationale of DO and pressure increases was to improve the oxygen availability and solubility, thus improving the driving force, ΔC, for the OTR. On the other hand, *k*_L_a can be increased by additions of salts or be decreased by antifoam additions [27]. In our production conditions, antifoam has only a single addition prior to the bioreactor inoculation. The salt feed starts with the fed-batch and continues to the end of the process. Although acceptable results in consistency and reproducibility could be obtained at both scales (Figure 4), some differences (Figure 5) still persisted due to the microorganism’s adaptive response to hypoxia.

### Exometabolomics or metabolite profiling of the small and large scale cultures

**Figure 11:**
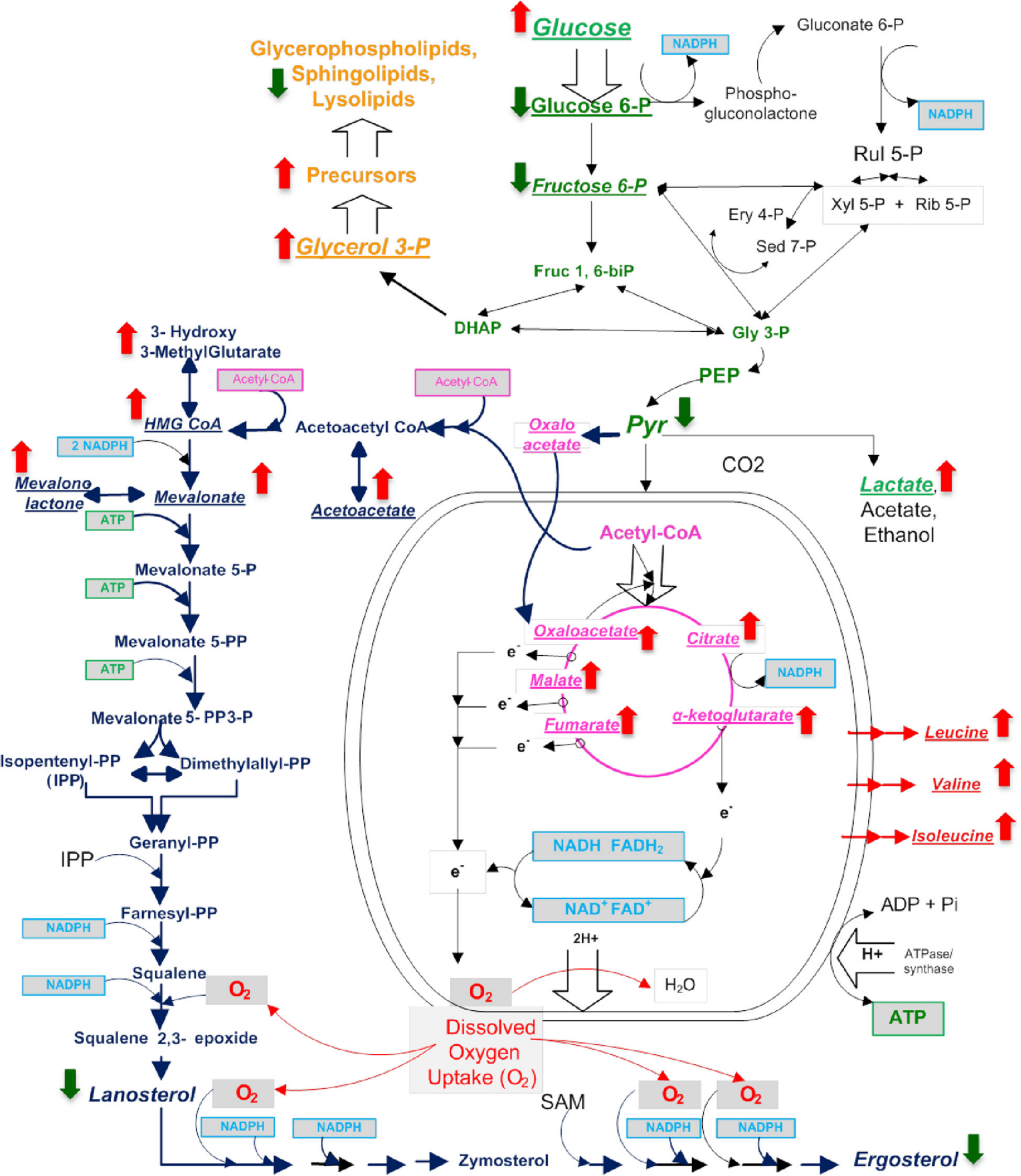
**Pathway diagram showing the summary of the exometabolomics related to the energy metabolism encompassing glycolysis, TCA cycle and mitochondrial respiration, and ergosterol and lipid synthesis.** Intermediates related to a particular pathway are color coded. Phosphorylation (ATP), redox (NAD(P)H), Acetyl-CoA intermediates/cofactors with high connectivity are also color coded. Red and green big arrows show changes in the 10,000 L scale with respect to that at the 10 L scale.

A comparative, semi-quantitative and time-dependent analysis of the exometabolome was performed in small and large scale bioreactors. This analysis revealed that several metabolites, including those constituting the central catabolic backbone of *S. cerevisiae*, i.e. glycolysis and TCA cycle, were excreted. A key observation is given by the apparent impairment of the TCA cycle in mitochondria due to hypoxic conditions in the large scale culture. The appearance of intermediates from the TCA cycle in the culture medium constitutes evidence of lack of electron flow through the respiratory chain, likely provoked by very low oxygen tensions (Figure 9). Thus, it stalls the flow through the TCA cycle eliciting intermediates accumulation followed by their release to the medium. At the large scale mitochondrial dysfunction would reverberate into upstream pathways producing overflow in glycolysis due to lack of pyruvate consumption by mitochondria; this would explain the increase in lactate and its excretion together with pyruvate (Figure 8). Glycolytic overflow can also explain the glucose shunt to alternative degradation pathways and the release of galactitol, erythrulose, gluconate into the culture medium (Additional file 1: Figure S1). Hypoxia phenomenon is also known to be at the origin of the diminished ergosterol and lipidic membrane components synthesis. These results indicate that prevailing oxygen limitation in the large scale high cell density cultures triggers mitochondrial dysfunction, eliciting overflow in upstream metabolic pathways such as glycolysis, flux redirection and impairment in lipid synthetic routes that depend on oxygen availability (Figure 11).

In our study the ergosterol precursors, 3-hydroxy-3-methylglutarate and acetoacetate (Figure 6A) and membrane precursors, choline, glycerol 3-phosphate, glycerophosphorylcholine (Figure 7A) were at higher level at 10,000 L scale than at 10 L scale, indicating higher membrane permeability at 10,000 L scale culture. However, the cell viability remained above 93% for both scales by the EOR under the production conditions, and the biomass measured by OD_600nm_ (data not shown) and DCW were comparable and kept on increasing to the EOR (Figure 4F), ruling out cell death/lysis. The results suggest there is no relationship between the appearance of membrane precursors and the cell viability. Higher level of membrane precursors at 10,000 L scale culture may be caused by the hypoxia conditions. From the changes detected by exometabolomics we conclude that the microorganism has truly adapted to the stress posed by the large scale conditions.

### Metabolic and physiological behavior of *S. cerevisiae* high cell density culture at the large scale

*S. cerevisiae* is a facultative aerobe that relies on mitochondrial respiration for generating energy in the form of electrochemical gradients and ATP (as illustrated in Figure 11). Additionally, in the mitochondria TCA cycle key precursors are generated for synthesis of sterol and fatty acids, which are main constituents of membrane assemblies, and affected by lack of oxygen. By transcriptomics and proteomics, *S. cerevisiae* at dissolved oxygen percentages of 0.5%, showed global up-regulation of genes encoding components of the respiratory pathway, and enzymes of TCA, resulting in a more efficient energy production [28]. The decrease in oxygen availability generates a large NAD(P)H pool that contributes to stalling metabolic fluxes due to diminished rate of pyridine nucleotides re-oxidation. This key event cascades in pathways up- and down-stream producing metabolites overflow and excretion. The TCA cycle and the mevalonate/ergosterol are among the major downstream pathways affected by low oxygen at the large scale (Figures 6, 9 and 11). Glycolysis and glucose shunt to alternative degradation pathways are among the major upstream affected pathways (Figures 8 and 11).

The diminished re-oxidation of NADH/FADH induces a TCA cycle overflow indicated by accumulation of oxaloacetate, malate, fumarate and citrate; these intermediates were significantly higher at 10,000 L than 10 L scale cultures (Figure 9). The lack of pyruvate oxidation into acetyl-CoA provoked further accumulation of lactate in the extracellular medium (Figure 8). Elevation of branched chain amino acids constitutes additional evidence of mitochondrial dysfunction (Figure 10). Epstein and coworkers [18] reported a comprehensive view of cellular responses to mitochondrial dysfunction using cDNA-based microarrays. Their results suggested that when part of the TCA cycle (succinate oxidation) cannot proceed in respiratory deficient cells, oxaloacetate and its condensation partner acetyl-CoA must be replenished stoichiometrically. Many of the up-regulated genes encoded for proteins that function: (*i*) in the conversion of metabolites generated from the β-oxidation of fatty acids into intermediates of TCA and glyoxylate cycles, such as acetyl-CoA, citrate, and succinate, (*ii*) in the conversion of pyruvate into oxaloacetate (by pyruvate carboxylase), (*iii*) in the conversion of branched-chain amino acids into acety-CoA and propionyl-CoA (by transaminases). Furthermore, this transcriptional reorganization to overcome blocks in the TCA cycle was associated with the lack of mitochondrial electron transport and therefore NADH re-oxidation depended on the activities for synthesis of glycerol-3-phosphate, lactate, acetate, and ethanol. Our results are in agreement, showing accumulation of metabolites like glycerol-3-phosphate (from glycolysis or reduced lipid synthesis), lactate (from glycolysis by overflow metabolism), valine, isoleucine, leucine (reduced branched chain amino acids catabolism), oxaloacetate, citrate, malate, fumarate, and precursor for lipid synthesis (choline, glycerophosphoethanolamine, glycerophosphorylcholine, and glycerol-3-phosphate). In particular, glycerol-3-phosphate and lactate accumulation indicate the presence of alternative mechanisms for re-oxidation of NADH to overcome the reduced mitochondrial electron transport by limited availability of oxygen and/or mitochondrial dysfunction (Figure 11).

Oxygen also intervenes directly in certain synthetic pathways. One such pathway in yeast is the mevalonate route that produces ergosterol. Thus, the observed metabolic effects provoked by hypoxia can be explained, at least in part, by the crucial role played by oxygen in ergosterol synthesis; this happens from geranyl-pyrophosphate (geranyl-PP), where four out of six redox reactions require oxygen (Figure 11). In the same process, NADPH is also consumed in several reactions. Additionally, 3 molecules of acetyl-CoA and ATP are required per molecule of ergosterol synthesized (Figure 11). Partial inhibition of ergosterol synthesis was suggested by increased 3-hydroxy-3-methylglutarate synthesis over time in the 10,000 L scale bioreactor. The change in the ergosterol synthesis influences membrane composition and permeability therefore affecting recombinant protein secretion and protein modification. Furthermore it has been demonstrated that mutants of *S. cerevisiae* defective in synthesis of ergosterol are viable but accumulate structurally altered sterols within the plasma membrane. Those results suggested that the membrane disorder and increasing occurrence of membrane voids within the plasmatic membrane synergistically enhance passive diffusion [29]. Clearly ergosterol plays an essential role in bulk membrane function, affecting membrane rigidity, fluidity, and permeability, so affecting endocytosis and membrane trafficking [29]. In addition, defective synthesis of sphingolipids and ergosterol impair the incorporation of Gas1p (a GPI-anchored α-1, 3-glucanosyltransglycosylate), to the cell wall [30]. Therefore the reduced Gas1p incorporation increased cell wall porosity due to reduced β-glucan crosslinking which might facilitate the passage of heterologous proteins. The consequences may include less stringent control of the exchange of macromolecules between cells and surrounding environment, possible including secreted proteins whose translocation through the cell is not affected by ergosterol and sphingolipid depletion [31].

The significant increase of the glycolytic intermediate glycerol-3-phosphate and lactate reflects metabolic overflow in the culture medium at 10,000 L (Figure 8). Glycerol-3-phosphate is a key precursor of phospho-, sphingo-, and lysolipids which can explain the changes in membrane lipid composition and permeability as reflected by the increase in WCW at the 10,000 L scale (Figure 5A).

Physiologically, microorganism yield on substrate (carbon and O_2_) and product is a main indicator of how efficiently coupled are the outcome (product and biomass) and the income (substrate utilization). In an industrial process, we seek to maintain high product yield that can be achieved by minimizing biomass and maximizing substrate utilization and its redirection to the product of interest. Thus, the metabolic/physiological efficiency for the microorganism is not equivalent to for the industrial objectives. Linton and Rye [32] reported data in the industrial production of exopolysaccharides, indicating that the highest rates of metabolite production occur in microorganisms possessing low efficiencies of energy conservation. It is of utmost industrial importance to maximize recoverable product per amount of substrate consumed by the host microorganism for cost-effective production. Consequently, understanding the bioprocess parameters that govern microbial growth and product yields is of fundamental importance for achieving the successful scale up.

Hypoxia was developed in the large scale bioreactor under the carbon-limiting fed-batch, high-cell density culture condition. As an indicator of the degree of coupling between respiration (catabolism) and energy conservation (anabolism), the decrease in *Y*_O2_ in the 10,000 L scale cultures revealed less biomass yield per mole of O_2_ consumed. When we correlated *Y*_O2_ to *q*_p_ and *Y*_p_, it was verified that lower *Y*_O2_ was accompanied by higher rate of production and level of the product (Table 1). Thus, at the 10,000 L scale, hypoxia triggers metabolic stress in *S. cerevisiae* that results in higher product yield.

A similar impact of hypoxia on the production of a human Fab fragment expressed in *Pichia pastoris* has been reported [31]. These authors attributed the improvement in product secretion to the impairment of cell membrane assembly. Other results regarding fed-batch like conditions for expression of Fab antibody fragment in *E. coli* indicated that oxygen-limited conditions promoted Fab accumulation into the extracellular medium [33]. Herein, in addition to the membrane impairment, we report that mitochondrial oxidative phosphorylation, as the primary target of hypoxia, triggers metabolic overflow decreasing the coupling between respiration and anabolism concomitantly with biomass yield, *Y*_O2_, but with a positive effect on product yield in the scaled up process.

## Conclusions

Collectively, we showed the successful scale-up of an optimized *S. cerevisiae* fermentation process from the laboratory to an industrial bioreactor. Major differences introduced by the 10,000 L scale that influenced the process controllability was DO that in turn affected mass transfer inside the bioreactor. The persistence of differences in apparent viscosity and wet cell weight, even after regaining control of the DO as one of the bioprocess parameters affected, led us to look into the impacts from the large scale conditions on the metabolic and physiological behavior of the host microorganism. The exometabolomics results indicate that reduced oxygen availability affected oxidative phosphorylation cascading into down- and up-stream pathways causing overflow metabolism. The study provides a striking example of the impact that stressful culture conditions at the industrial scale may exert on the control and performance of a bioprocess. Also the metabolic/physiological efficiency for the microorganism is not equivalent to the industrial objectives. Consequently, understanding the bioprocess parameters that govern microbial growth and product yields is of fundamental importance for achieving the successful scale up.

## Methods

### Strain and fermentation process

The *S. cerevisiae* strain was originally developed from the parental *S. cerevisiae* strain AH22 (ATCC 38626) and the gene encoding for the product is in a high-copy plasmid. The fermentation process to produce a therapeutic recombinant protein, Pr-1, at the small scale (10 L) was described previously [17]. Briefly, Pr-1 production is conditionally induced in the host strain by glucose limitation that elicits product secretion into the medium. The fermentation process starts with the inoculation of a vial from a working cell bank into a 3 L disposable baffled Erlenmeyer shake flask (Corning, MA, US) containing 1 L of defined shake flask medium. This medium was modified from the buffered minimal medium (BMM) as described by Goodey *et al.*[34]. The culture was incubated and agitated in a rotary shaker at 30°C and 225 rpm until glucose was depleted. The flask culture was then used to inoculate a 10 L seed bioreactor (Biolafitte, Saint Germain en Laye, France) to achieve higher cell density. The seed medium was similar to the production medium with the exception of removal of Tween 80. Both the batch and feed media were modified from the MW10 medium as described by Goodey *et al.*[34]. The modified MW10 feed media were separated with a glucose nutrient feed and a one-molar (1 M) phosphate salt solution feed to minimize the salt precipitation. The seed and production bioreactors were controlled by the Distributed Control System (Siemens Moore APACS, US). DO is controlled at desired set points (30% at the seed bioreactor, and 12.5% at the production bioreactor) by a cascade system of automatically first ramping up agitation until a maximum (900 rpm), and continues, if necessary, by injecting pure oxygen being blend into the inflowing air fixed at a total gas sparge rate. The pH was controlled at 6.0 and 5.75, for the seed and production bioreactor, respectively, using ammonium hydroxide (30%) and phosphoric acid (17%) additions. The temperature was controlled at 29°C. The backpressure for the seed bioreactor was at 7 psi. The backpressure for 10 L production cultures was initially at 5 psi and after the scale-up was increased to 10 psi as set up at 10,000 L scale conditions. The seed expansion consisted of a batch phase followed by a fed-batch phase. Once the target optical density (OD_600_) of 100 ± 20 was reached, a portion of the culture was used to inoculate a 10 L production bioreactor with a target inoculation of OD_600_ 15. Both a glucose nutrient feed and a phosphate salt solution feed were initiated when glucose concentration was ≤ 0.3 g/L in the batch medium. Glucose concentration was measured offline with a YSI biochemistry analyzer (YSI, OH, US). OD_600_, WCW, DCW and various other metabolites such as ethanol and acetate were also monitored. The fermentation process was terminated after 80 hrs of fed-batch time. Fermentation supernatant samples were collected for checking product quantity and quality, and metabolite profiling.

### Scale-up fermentation process

For scaling-up to a 10,000 L scale bioreactor, a full seed train was implemented, including shake flask cultures and seed bioreactors. The seed bioreactor culture was used to inoculate the 10,000 L scale bioreactor when the cell density reached the target OD_600_ of 100 ± 20. The same seed cultures were also used to inoculate runs 9–16 at the 10 L scale production bioreactor. The scale-independent process parameters such as temperature, pH, DO and backpressure were kept the same as in the 10 L reactors. The backpressure for production bioreactor was initially started at 5 psi and increased to 10 psi. The DO was initially started at 12.5%, and increased to 25% during the scale-up. The scale-dependent process parameters such as batch volume, glucose nutrient and phosphate salt feed rates as well as total gas flow rate were accordingly scaled. The fermentation process was terminated after 80 hrs of fed-batch time. Fermentation supernatant samples were collected for analysis of product quantity and quality, and metabolite profiling.

### Determination of mass transfer coefficient and oxygen uptake rate

The exhaust gas components were measured online by mass spectrometer (Model: Prima dB, Thermo Electron Corporation, Huston, US). Oxygen uptake rate (OUR) is determined via mass spectrometer using measurements of O_2_ percent in the inlet and outlet gas, gas flow rates and broth volume according to the following formulas:

The total inflowing gas rate is the sum of all flow rates which pass through the mass flow controllers operated by the Distributed Control System, including air and oxygen measured in slpm. The total inflowing gas rate is thus determined by Equation 1:

(1) $$F_{\text{TOT}}=F_{\text{AIR}}+F_{O2}$$

The quantities in millimoles (mmol) per hour of oxygen and carbon dioxide going in were calculated using Equations 2 and 3. The pure oxygen supply is assumed to be 100% oxygen. The composition of the air supply is measured by the mass spectrometer and reported in percent. These percentages are converted into fractions by dividing by 100. Taking the fraction of oxygen in the air (ppO2_AIR_ as a fraction 0 to 1) and the fraction of CO_2_ in the air (ppCO_2AIR_ as a fraction 0 to 1) to compute:

(2) $$O2_{\text{IN}}=\frac{\left(F_{O2}+\left(F_{\text{AIR}}*\text{ppO}2_{\text{AIR}}\;\right)\right)}{22.4}*1000*60$$

(3) $$\text{CO}2_{\text{IN}}=\frac{\left(F_{\text{AIR}}*\text{ppCO}2_{\text{AIR}}\;\right)}{22.4}*1000*60$$

The flows *F*_*O2*_ and *F*_*AIR*_ are in slpm, there are 22.4 standard liters per mole of any ideal gas at 273 K and 1 atm; the equivalencies of 1000 millimoles per mole as well as 60 min/hr obtain the mmol/hr.

The quantities of oxygen and carbon dioxide coming out are estimated by applying Equations 4 and 5. The volume of exhaust gas in slpm is assumed to be equal to the total volume going in. The composition of the exhaust gas is measured by the mass spectrometer. Taking the fraction of oxygen in the exhaust (ppO_2EXH_ as a fraction 0 to 1) and the fraction of CO_2_ in the exhaust (ppCO_2EXH_ as a fraction 0 to 1) to compute:

(4) $$O2_{\text{OUT}}=\frac{\left(F_{\text{TOT}}*\text{ppO}2_{\text{EXH}}\;\right)}{22.4}*1000*60$$

(5) $$\text{CO}2_{\text{OUT}}=\frac{\left(F_{\text{TOT}}*\text{ppCO}2_{\text{EXH}}\;\right)}{22.4}*1000*60$$

The equivalencies of 1000 millimoles per mole and 60 min/hr are the units in mmol/hr.

OUR and carbon dioxide evolution rates (CER) are normalized by the broth volume as shown in Equations 6 and 7:

(6) $$\text{OUR}=\frac{\left(O2_{\text{IN}}-O2_{\text{OUT}}\right)}{\text{Vo}l_{\text{Broth}}}$$

(7) $$\text{CER}=\frac{\left(\text{CO}2_{\text{OUT}}-\text{CO}2_{\text{IN}}\right)}{\text{Vo}l_{\text{Broth}}}$$

Since the gas quantities are in mmol/hr and broth volume is in Liters, the units of OUR and CER are mmol/L/hr. In addition, the respiratory quotient (RQ) follows the Equation 8:

(8) $$\text{RQ}=\frac{\text{CER}}{\text{OUR}}$$

At steady state operation, the OTR from the gaseous phase to the liquid phase in a bioprocess is equal to the cellular OUR. Given that OUR is measured directly via mass spectrometry, the volumetric mass transfer coefficient *k*_*L*_*a* is defined by Equation 9:

(9) $$k_{L}a=\frac{\text{OUR}}{\left(C_{O2}^{*}-C_{L}\right)}=\frac{\text{OUR}}{\mathrm{\Delta C}}$$

The *k*_*L*_*a* in hr^−1^ was converted to min^−1^.

The following Equation 10 shows the determination of *ΔC* in mmol/L:

(10) $$\mathrm{\Delta C}=\frac{\left(C_{\text{spg}}^{\text{IN}}-C_{\text{surf}}^{\text{OUT}}\right)}{\text{Ln}\frac{\left(C_{\text{spg}}^{\text{IN}}-C_{L}\right)}{\left(C_{\text{surf}}^{\text{OUT}}-C_{L}\right)}}$$

Considering 21% percent of oxygen in the air ($O2_{\text{percent}}^{\text{IN}}$), and assuming the volume of exhaust gas to be equal to the total volume going in (which is correct on average), every term of concentration (*C*), in mmol/L, is determined by Equations 11, 12 and 13:

(11) $$C_{\text{spg}}^{\text{IN}}=\frac{\left(F_{\text{AIR}}*O2_{\text{percent}}^{\text{IN}}+F_{O2}\right)}{F_{\text{TOT}}}*O2_{\text{Sol}}=\text{ppO}2_{\text{IN}}*C_{O2}^{*}$$

(12) $$C_{\text{surf}}^{\text{OUT}}=O2_{\text{percent}}^{\text{OUT}}*O2_{\text{Sol}}=\text{ppO}2_{\text{OUT}}*C_{O2}^{*}$$

(13) $$C_{L}=DO_{\text{percent}}^{\text{NET}}*O2_{\text{Sol}}=\text{ppO}2_{\text{NET}}*C_{O2}^{*}$$

Where:

$O2_{\text{percent}}^{\text{OUT}}$ = oxygen percent in exhaust by mass spectrometer

*F*_*O*2_ = moving average volumetric flow rate of pure oxygen in slpm

*F*_*AIR*_ = moving average volumetric flow rate of air in slpm

*C*_*L*_ = the net dissolved oxygen (DO) concentration in the liquid phase

*O*2_*Sol*_ = dissolved oxygen concentration in water under the studied pressure and temperature, also known as oxygen solubility ($C_{O2}^{*}$)

The logarithmic average value between the inlet and outlet gas streams was used to calculate the approximate driving force because the variation in oxygen transfer driving force ΔC can be significant within a large scale bioreactor.

### Analytical methods

Quantification of protein Pr-1 was performed as before [17]. Broth titer was calculated using the supernatant titer corrected with WCW. For WCW determination, 10 mL of broth were centrifuged at 10,400 RCF for 35 min; the supernatant was decanted. The resulted pellet weight was then divided by the weight of original broth to get a wet weight value expressed as g/kg of broth. For DCW determination, 1 mL of broth was centrifuged at 13,000 RCF for 5 min. The supernatants were collected as spent medium for further analysis. The pellet obtained was washed, dried for approximately 18 hr at 95°C, and the dried pellet weight was divided by the weight of original broth to get a DCW value expressed as g/kg of broth.

Culture apparent viscosity was measured using a Brookfield cone and plate viscometer HA DVII + Pro (Middleboro, MA) according to the manufacturer’s manual. The measurement was done with a cone and plate CPE-40 sample cup and spindle at 30, 60 and 100 rpm to assess the culture rheology characteristics at room temperature. The shear rate was calculated based on the rotation speed (rpm) according to: Shear rate (s^−1^) = 7.5* N where N is the spindle rotational speed. 30, 60 and 100 rpm corresponded to shear rates of 225, 450 and 750 s^−1^, respectively. The apparent viscosity was determined from a single point at shear rate of 750 (s^−1^) at room temperature. The resulting apparent viscosity was reported in centipoises (cP) (as reference for equivalency with Pascal seconds, 1 cP = 1 mPa · s = 0.001 Pa · s).

The *S. cerevisiae* cell viability was measured using a BD FACS Calibur Flow Cytometer (BD Bioscience, San Jose, CA, US) as described in Garcia *et al*., [35] and the manufacturer’s instruction. Samples taken from the culture were immediately diluted with Phosphate Buffered Saline (PBS; pH 7.2) to a final concentration of ~10^6^ cells mL^−1^ and stained with the red fluorescence dye, propidium iodide (PI). This fluorophore cannot penetrate the live *S. cerevisiae* cell membrane, but can penetrate the compromised membranes of nonviable cells. The cell viability was reported as the percentage of the total measured cells.

### Metabolic profiling

#### Study design

The samples were from 10 L scale identified as runs 9 to 13 and from 10,000 L scale identified as batches 4 to 8. A total of five media replicates (or spent media) for each scale were collected at EFT 46, 54, 64, 76 and 80 hr, and used for metabolite profiling at Metabolon, Inc. (Durham, NC, US). Additionally, one batch medium sample and one glucose feed sample were included for reference. In total, 52 samples representing 5 time points for 2 reactor scales (n = 5 each), plus the two reference samples were analyzed.

#### Sample preparation

Spent medium samples were thawed on ice, and a 100 μL volume sample was used for extraction. Extraction was performed by adding 450 μL of methanol, containing recovery standards with vigorous shaking for 2 min using Glen Mills GenoGrinder 2000 (Ops Diagnostics, NJ). The samples were then centrifuged, supernatant removed (MicroLab STAR® robotics), and placed briefly on a TurboVap® (Zymark) to remove the organic solvent. Each sample was dried under vacuum overnight. Samples were then prepared for the appropriate instrument, either liquid chromatography/mass spectrometry/mass spectrometry (LC/MS/MS) for basic species and acidic species, or gas chromatography/mass spectrometry (GC/MS).

#### Liquid chromatography/mass spectrometry and gas chromatography/mass spectrometry

The LC/MS portion of the platform incorporated a Waters Acquity UHPLC system and a Thermo-Finnigan LTQ mass spectrometer, including an electrospray ionization source and linear ion-trap mass analyzer. Aliquots of the vacuum-dried samples were reconstituted; one each in acidic or basic LC-compatible solvents contains 8 or more injection standards at fixed concentrations (to both ensure injection and chromatographic consistency). Extracts were loaded onto columns (Waters UHPLC BEH C18-2.1 × 100 mm, 1.7 μm) and gradient-eluted with water and 95% methanol containing 0.1% formic acid (acidic extracts) or 6.5 mM ammonium bicarbonate (basic extracts). Samples for GC/MS analysis were dried under vacuum desiccation for a minimum of 18 hrs prior to being derivatized under nitrogen using bistrimethyl-silyl-trifluoroacetamide. The GC column was 5% phenyl dimethyl silicone and the temperature ramp was from 60°C to 340°C over a 17-minute period. All samples were then analyzed on a Thermo-Fisher Scientific Trace DSQ fast-scanning single-quadrupole mass spectrometer using electron ionization. Chromatographic separation followed by full-scan mass spectra was carried out to record retention time and molecular weight (m/z) of all detectable ions presented in the samples. Additionally, on the two LC methods (which are capable of tandem mass spectrometry), retention time, molecular weight (m/z), and MS/MS^2^ fragmentation data of all detectable ions were recorded.

#### Compound identification, quantification, and data curation

Metabolites were identified by automated comparison of the ion features in the experimental samples to a reference library of over 3000 purified, authenticated chemical standard entries that include retention time, molecular weight (m/z), preferred adducts, and in-source fragments as well as their associated MS/MS^2^ spectra. In brief, samples were extracted and split into equal parts for analysis on the GC/MS and two LC/MS/MS platforms. Proprietary software was used to match ions to the in-house library of standards for metabolite identification and for metabolite quantitation by peak area integration.

#### Data collection, normalization, and visualization

A client matrix composed of small aliquots of all sample extracts in this study was created. The client matrix technical replicate samples were treated independently throughout the process. All process samples (client matrix, and a mixture of organic components used to assess GC column performance, process blanks, etc.) were spaced evenly among the injections for each day and all samples were randomly distributed throughout each day’s run. Data were collected over multiple platform run days and thus, ‘block normalized’ by calculating the median values for each run-day block for each individual compound. This minimizes any inter-day instrument gain or drift, but does not interfere with intra-day sample variability. Missing values (if any) were assumed to be below the level of detection for that biochemical with the instrumentation used and were imputed with the observed minimum for that particular biochemical.

For visualization of biochemical differences between the various treatment groups, the data are displayed in line plot graph format. The data selected for display by line plot were filtered by statistics or included for completion of a biochemical pathway. An interpretive guide for this display is shown in Additional file 2: Figure S2A.

#### Data statistical analyses

Biochemical data were analyzed by Welch’s two-sample *t*-tests to test that the means of two independent groups are equal. The heat map tables are color coded, and descriptions are shown in Additional file 2: Figure S2B. Often Welch’s correction was applied to allow for unequal variances between the groups. The *p*-value relates the probability of obtaining a result as or more extreme than the observed data; a low *p*-value (p ≤ 0.05) is generally accepted as strong evidence that the two means are different. The *q*-value describes the false discovery rate; a low *q*-value (*q* ≤ 0.10) is an indication of high confidence in a result.

In the case of the pathway heat map table, this format is associated with the statistical analysis of the data. Statistical cut-offs are typically employed for determining candidate metabolites that might be physiologically significant in a given study. The relatively conservative criteria of *p* ≤ 0.05 and *q* ≤ 0.10 are routinely used in metabolomic studies, which allows for the identification of metabolites that are significantly altered in response to a treatment or significantly different between two comparison groups and would be expected to yield a false discovery rate of no more than 10%. For the purposes of this study, comparisons between the various treatment groups were taken as significant when *p* ≤ 0.05, regardless of the *q*-value. This approach was taken in order to be more inclusive of data that otherwise might not meet the strict statistical cut-off values.

## Abbreviations

CER: Carbon dioxide evolution rate; CPP: Critical process parameter; DCW: Dry cell weight; DO: Dissolved oxygen; EFT: Elapsed fermentation time; EOR: End of run; kLa: Mass transfer coefficient; OUR: Oxygen uptake rate; OTR: Oxygen transfer rate; RQ: Respiratory quotient; slpm: Standard liter per minute; TCA: Tricarboxylic acid; WCW: Wet cell weight; L: Liter; Ys: Biomass yield based on carbon substrate (glucose) fed (g DCW/g Gluc); Yp: Product yield based on carbon substrate (glucose) fed (mg Pr-1/mol Gluc); qp: Specific rate of product (protein Pr-1) production per gram of glucose fed and per time (mg Pr-1/g Gluc/hr); YO2: Biomass yield based on molecular oxygen consumed by cells (g DCW/mol O_2_).

## Competing interests

The authors declare that they have no competing interests.

## Authors’ contributions

ZF carried out fermentation batches, collected samples from both scales, data analysis, and drafted the manuscript. JL carried out fermentation batches, collected samples and data from lab scale. BS carried out exometabolomics data mining and interpretation, and manuscript revision. TV carried out data mining, quantification of mass transfer at the large and lab scales, and manuscript revision. EA and PP participated in discussions for interpretation of results, and manuscript revision. JCA conceived this study, participated in the interpretation of the results, and assisted in the manuscript drafting. All authors read and accepted the final manuscript.

## Supplementary Material

Additional file 1: Figure S1 Line plot graphs showing the differences in glucose metabolism between scales. Red and green arrows show changes in the 10,000 L scale with respect to that at the 10 L scale. The intermediates measurement and data analysis were determined as described in “Methods”. Error bars represent “mean +/− one standard deviation”. Click here for file

Additional file 2: Figure S2 Visualization of the metabolite profiling data in line plot graph and the heat map table. Data are scaled such that the median value measured across all samples was set to 1.0. A) Line plot graph. The data for each bioreactor scale is designated as shown (10 L = **green line**, 10,000 L = **yellow line**, basal media “M” = **blue point**, feed media “F” = **grey point**). B) Heat map table analysis. Error bars represent “mean +/− one standard deviation”. Click here for file

## References

[B1] Ferrer-Miralles N Domingo-Espin J Corchero JL Vazquez E Villaverde A Microbial factories for recombinant pharmaceuticals Microb Cell Fact 2009 8 17 24 10.1186/1475-2859-8-17 19317892 PMC2669800

[B2] Holmes WJ Darby RAJ Wilks MDB Smith R Bill RM Developing a scalable model of recombinant protein yield from *Pichia pastoris*: the influence of culture conditions, biomass and induction regime Microb Cell Fact 2009 8 35 48 10.1186/1475-2859-8-35 19570229 PMC2717918

[B3] Siurkus J Panula-Perala J Horn U Kraft M Rimseliene R Neubauer P Novel approach of high cell density recombinant bioprocess development: optimization and scale-up from microlitre to pilot scales while maintaining the fed-batch cultivation mode of *E. coli* cultures Microb Cell Fact 2010 9 35 51 10.1186/1475-2859-9-35 20487563 PMC2890543

[B4] Amanullah A BuckLand BC Nienow AW Paul EL, Atiemo-Obeng VA, Kresta SM Mixing in the fermentation and cell culture industries Handbook of Industrial Mixing: Science and Practice 2003 New York: John Wiley & Sons 1071 1170

[B5] Xu B Jahic M Blomsten G Enfors SO Glucose overflow metabolism and mixed-acid fermentation in aerobic large-scale fed-batch processes with *Escherichia coli* Appl Microbiol Biotechnol 1999 51 564 571 10.1007/s002530051433 10390814

[B6] Vrabel P van der Lans RGJM Luyben KCAM Boon LA Nienow AW Mixing in large-scale vessels, stirred with multiple radial and axial pumping up impellers: modeling and measurements Chem Eng Sci 2000 55 5881 5896 10.1016/S0009-2509(00)00175-5

[B7] Enfors SO Jahic M Rozkov A Xu B Hecker M Jurgen B Kruger E Shweder T Hamer G O’Beirne D Noisommit-Rizzi N Reuss M Boone L Hewitt C McFarlane C Nienow A Kovacs T Trägårdh C Fuchs L Revstedt J Friberg PC Hjertager B Blomsten G Skogman H Hjort S Hoeks F Lin HY Neubauer P van der Lans R Luyben K Vrabel P Manelius A Physiological responses to mixing in large bioreactors J Biotechnol 2001 85 175 185 10.1016/S0168-1656(00)00365-5 11165362

[B8] Bylund F Collet E Enfors SO Larsson G Substrate gradient formation in the large-scale bioreactor lowers cell yield and increases by-product formation Bioproc Eng 1998 18 171 180 10.1007/s004490050427

[B9] Oosterhuis NMG Kossen NWF Olivier APC Schenk ES Scale-down and optimization studies of the gluconic acid fermentation by *Gluconobacter oxydans* Biotechnol Bioeng 1985 27 711 720 10.1002/bit.260270521 18553727

[B10] Amanullah A Christensen LH Hansen K Nienow AW Thomas CR Dependence of morphology on agitation intensity in fed-batch cultures of *Aspergillus oryzae* and its implications for recombinant protein production Biotechnol Bioeng 2002 77 815 826 10.1002/bit.10181 11835142

[B11] Riley GL Tucker KG Paul GC Thomas CR Effect of biomass concentration and mycelial morphology on fermentation broth rheology Biotechnol Bioeng 2000 68 160 172 10.1002/(SICI)1097-0290(20000420)68:2<160::AID-BIT5>3.0.CO;2-P 10712732

[B12] Junker BH Scale-up methodologies for *Escherichia coli* and yeast fermentation processes J Biosci Bioeng 2004 97 347 364 10.1016/S1389-1723(04)70218-2 16233642

[B13] Hewitt CJ Nebe-Von Caron G Axelsson B McFarlane CM Nienow AW Studies related to the scale-up of high-cell-density *E. coli* fed-batch fermentations using multiparameter flow cytometry: effect of changing microenvironment with respect to glucose and dissolved oxygen concentration Biotechnol Bioeng 2000 70 381 390 10.1002/1097-0290(20001120)70:4<381::AID-BIT3>3.0.CO;2-0 11005920

[B14] Nienow AW Hydrodynamics of stirred bioreactors App Mech Rev 1998 51 3 32 10.1115/1.3098990

[B15] Schweder T Krüger E Xu B Jürgen B Blomsten G Enfors SO Hecker M Monitoring of genes that respond to process-related stress in large-scale bioprocesses Biotechnol Bioeng 1999 65 151 159 10.1002/(SICI)1097-0290(19991020)65:2<151::AID-BIT4>3.0.CO;2-V 10458735

[B16] Paczia N Nilgen A Lehmann T Gatgens J Wiechert W Noack S Extensive exometabolome analysis reveals extended overflow metabolism in various microorganism Microb Cell Fact 2012 11 122 136 10.1186/1475-2859-11-122 22963408 PMC3526501

[B17] Fu Z Leighton J Cheng A Appelbaum E Aon JC Optimization of a *Saccharomyces cerevisiae* fermentation process for production of a therapeutic recombinant protein using a multivariate Bayesian approach Biotechnol Prog 2012 28 1095 1105 10.1002/btpr.1557 22581591

[B18] Epstein CB Waddle JA Hale W IV Dave V Thornton J Macatee TL Garner HR Butow RA Genome-wide responses to mitochondrial dysfunction Mol Biol Cell 2001 12 297 308 10.1091/mbc.12.2.297 11179416 PMC30944

[B19] Valerio A D’Antona G Nisoli E Branched-chain amino acids, mitochondrial biogenesis, and healthspan: an evolucionary perspective Aging 2011 3 464 478 21566257 10.18632/aging.100322 PMC3156598

[B20] Linton JD The relationship between metabolite production and the growth efficiency of the producing organism FEMS Microbiol Rev 1990 75 1 18 10.1111/j.1574-6968.1990.tb04083.x 2186758

[B21] Enfors SO Enfors SO Preface Advances in Biochemical Engineering/Biotechnology-Physiological Stress Responses in Bioprocesses 2004 New York: Springer 89

[B22] Soini J Ukkonen K Neubauer P High cell density media for *Escherichia coli* are generally designed for aerobic cultivations - consequences for large-scale bioprocesses and shake flask cultures Microb Cell Fact 2008 7 26 36 10.1186/1475-2859-7-26 18687130 PMC2526063

[B23] Wick LM Egli T Enfors SO Molecular components of physiological stress responses in *E. coli* Advances in Biochemical Engineering/Biotechnology-Physiological Stress Responses in Bioprocesses 2004 New York: Springer 89: 1-46 10.1007/b93957 15217154

[B24] Malinowski JJ Lafforgue L Goma G Rheological behavior of high density continuous cultures of *Saccharomyces cerevisiae* J Ferment Technol 1987 65 319 323 10.1016/0385-6380(87)90094-X

[B25] Bhargava S Wenger KS Marten MR Pulsed feeding during fed-batch *Aspergillus oryzae* fermentation leads to improved oxygen mass transfer Biotechnol Prog 2003 19 1091 1094 10.1021/bp025694p 12790687

[B26] Tuffile CM Pinho F Determination of oxygen transfer coefficients in viscous streptomycete fermentations Biotechnol Bioeng 1970 12 849 871 10.1002/bit.260120602

[B27] Nienow AW Nienow AW Aeration-Biotechnology Kirk Othmer Encyclopedia of Chemical Technology 2003 New York: John Wiley & Sons 1: 730-747

[B28] Rintala E Toivari M Pitkanen JP Wiebe M Ruohonen L Penttila M Low oxygen levels as a trigger for enhancement of respiratory metabolism in *Saccharomyces cerevisiae* BMC Genomics 2009 10 461 480 10.1186/1471-2164-10-461 19804647 PMC2767370

[B29] Abe F Hiraki T Mechanistic role of ergosterol in membrane rigidity and cycloheximide resistance in *Saccharomyces cerevisiae* Biochim Biophys Acta 2009 1788 743 752 10.1016/j.bbamem.2008.12.002 19118519

[B30] Bagnat M Keranen S Shevchenko A Simons K Lipids rafts function in biosynthetic delivery of proteins to the cell surface in yeast Proc Natl Acad Sci U S A 2000 97 3254 3259 10.1073/pnas.97.7.3254 10716729 PMC16225

[B31] Baumann K Adelantado N Lang C Mattanovich D Ferrer P Protein trafficking, ergosterol biosynthesis and membrane physics impact recombinant protein secretion in *Pichia pastoris* Microbiol Cell Fact 2011 10 93 108 10.1186/1475-2859-10-93 PMC3219557 22050768

[B32] Linton JD Rye AJ The relationship between energetic efficiency in different microorganisms and the rate and type of metabolite over-produced J Ind Microbiol 1989 4 85 96 10.1007/BF01569792

[B33] Ukkonen K Veijola J Vasal A Neubauer P Effect of culture medium, host strain and oxygen transfer on recombinant Fab antibody fragment yield and leakage to medium in shaken *E. coli* cultures Microb Cell Fact 2013 12 73 86 10.1186/1475-2859-12-73 23895637 PMC3733871

[B34] Goodey AR Sleep D van Urk H Berezenko S Woodrow JR Johnson RA Wood PC Burton SJ Quirk AV Coghlan DJ Wilson MJ High purity albumin and method of producing 1998 Patent US 5,728,553

[B35] Garcia MT Sanz R Galceran MT Puignou L Use of fluorescent probes for determination of yeast cell viability by gravitational field-flow fractionation Biotechnol Prog 2006 22 847 852 10.1021/bp050421q 16739970

