# Supplementary material for: Exometabolome analysis reveals hypoxia at the up-scaling of a Saccharomyces cerevisiae high-cell density fed-batch biopharmaceutical process
Source: Microb Cell Fact. 2014 Mar 5;13:32. doi: 10.1186/1475-2859-13-32 (PMC4016033; doi:10.1186/1475-2859-13-32)
Supplement: Additional file 2: Figure S2 — Visualization of the metabolite profiling data in line plot graph and the heat map table. Data are scaled such that the median value measured across all samples was set to 1.0. A) Line plot graph. The data for each bioreactor scale is designated as shown (10 L = green line, 10,000 L = yellow line, basal media “M” = blue point, feed media “F” = grey point). B) Heat map table analysis. Error bars represent “mean +/− one standard deviation”. [file 1475-2859-13-32-S2.docx]

Additional file 2: Figure S2

A)

B)
